# Selection of semiconducting metal oxides for light-activated gas sensors at low temperature

**DOI:** 10.1007/s00216-026-06713-w

**Published:** 2026-09-02

**Authors:** Benjamin Junker-Reiss, Carolin Ewald, Peter Janoschek, Xiao-Xue Wang, Malte Schalk, Udo Weimar, Lutz Mädler, Nicolae Bârsan

**Affiliations:** 1 Institute of Physical and Theoretical Chemistry, University of Tübingen, Auf der Morgenstelle 15, 72076 Tübingen, Germany; 2 Center for Light-Matter Interaction, Sensors and Analytics (LISA+), University of Tübingen, Auf der Morgenstelle 15, 72076 Tübingen, Germany; 3 Leibniz Institute for Materials Engineering, University of Bremen, Badgasteiner Strasse 3, 28359 Bremen, Germany; 4 Faculty of Advanced Science and Technology (FAST), Kumamoto University, 2-39-1 Kurokami, Kumamoto, 860-8555 Kumamoto Japan

**Keywords:** Gas sensors, Light activation, Band gap, Flame spray pyrolysis, Semiconducting metal oxide, Long-term stability

## Abstract

**Abstract:**

Light activation is a promising approach for lowering the power consumption and increasing the selectivity of chemoresistive gas sensors based on semiconducting metal oxides. In this study, different aspects of commonly used materials are investigated, compared, and evaluated for their application as gas sensors. Their ability to detect CO and NO$_{2}$ at low temperature (70 $^{\circ }$C) highly depends on the wavelength used for activation. The reaction with atmospheric oxygen is also highly affected by illumination, as adsorption, desorption, and free charge carrier concentration are influenced by light. For this reaction, the relationship between band gap and photon energy becomes apparent, which is not the case for the detection of analyte molecules. Investigations on the long-term stability indicate that only certain combinations of wavelengths and materials are suitable for prolonged and stable operation.

**Graphic abstract:**

**Figure:**
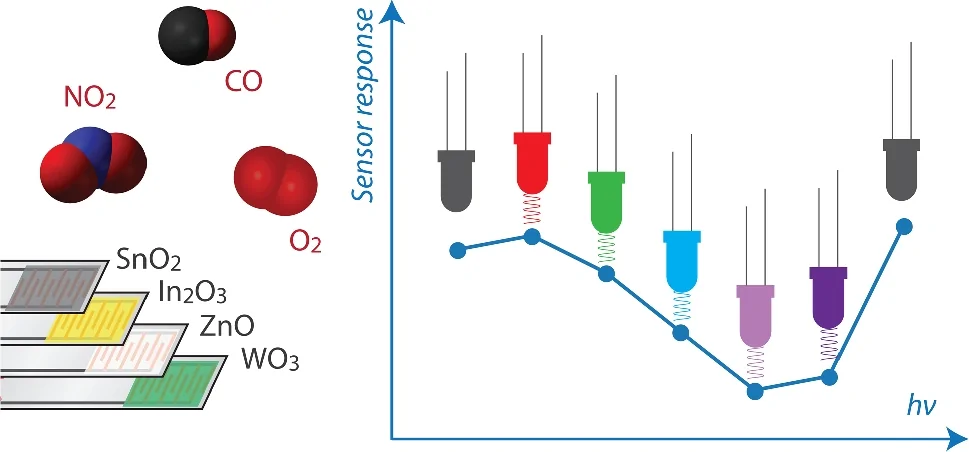

## Introduction

**Table 1:** Reagents and parameters used for the direct deposition of sensing layers via FSP

Material	Solvate	Concentration	Solvent	Volume
		mol/l		ml
WO$_{3}$	Tungsten hexacarbonyl	0.1	Tetrahydrofuran	100
In$_{2}$O$_{3}$	Indium acetylacetonate	0.1	Xylene	100
SnO$_{2}$	Tin 2-ethyl-hexanoate	0.5	Xylene	25
ZnO	Zinc naphtenate	0.5	Xylene	100
CuO	Copper naphthenate	0.1	Xylene	100
Co$_{3}$O$_{4}$	Cobalt naphthenate	0.1	Xylene	100
BiVO$_{4}$	Bismuth neodecanoate, vanadium(V) oxytripropoxide	0.125	Xylene	100

Numerous applications require the detection of gaseous analytes in the atmosphere. Protecting people from pollutants and odors using intelligent ventilation controls in buildings and cars as well as the detection of noxious substances in workplaces can be provided by the same technology [1–3]. While for many industrial applications advanced sensing technologies, e.g., electrochemical cells or optical detectors, are applied, the needs for gas sensors in everyday appliances have different requirements: Sensors must be versatile, cheap, small, and low in consumption of energy [4–9]. All of these requirements are met to great extent by chemoresistive gas sensors based on semiconducting metal oxides (SMOX). However, a lack of selectivity is considered one of their major drawbacks [3, 10]. Strategies to mitigate this include the development of new materials, e.g., by nanostructuring or noble metal loadings, the combination of multiple sensors into an array, or temperature-modulated operation [11–14]. Another promising and yet not broadly applied strategy is to utilize light instead of heat to provide the activation for chemical reactions between the analytes and the sensor’s surface [15, 16]. Not only will this help in decreasing their power consumption, but it also allows the tuning of the sensors’ selectivity.

While the first reports on the detection of gases with SMOX date back to 1957 [17], studies on the photoconductivity of semiconductors and adsorption of oxygen have been performed earlier [18, 19]. Despite these efforts, light-activated gas sensors received only little attention in the next decades, despite the advent of LEDs as small and efficient light sources. Even in the 1990s, high-power UV lamps were used to operate SnO$_{2}$ gas sensors at room temperature [20–22]. Nowadays, it is well known that light activation at low temperature (usually between room temperature and 100 $^{\circ }$C) is also possible with photons of energy lower than the semiconductor’s band gap, and therefore, cheaper and more efficient LEDs in the visible range can be used [16].

In a previous study, the performance of four different SMOX materials was compared—with special focus on their individual dependency on wavelength [23]. Each of the materials was affected differently by the illumination, with WO$_{3}$ having the lowest and SnO$_{2}$ the highest dependency on wavelength. Furthermore, more detailed studies on the effect of illumination on WO$_{3}$ have been performed using operando diffuse reflectance infrared Fourier transformation spectroscopy (DRIFTS) [24]. The aforementioned possibility to activate gas sensors with photons of energy below the SMOX band gap could be confirmed spectroscopically [25].

The selection of materials for this study was extended to include the low band gap materials C$_{\text {u}}$O, Co$_{3}$O$_{4}$, and BiVO$_{4}$. All materials were produced by direct deposition onto sensor substrates using flame spray pyrolysis (FSP). This offers the advantage of producing highly porous layers of quasi-spherical primary particles of uniform size [26, 27]. As a consequence, the sensing layers exhibit a high specific surface area and, due to the low density, uniform and high irradiance throughout their cross section.

The materials’ sensing performances towards a reducing (CO) and an oxidizing (NO$_{2}$) gas are investigated. In addition, the interplay of illumination and oxygen at the sensor surface is investigated since oxygen is an omnipresent compound and accompanies the sensing process of each analyte. Finally, one of the most critical aspects for application, long-term stability, is investigated for the most relevant materials. The goal is to provide general guidelines for the selection of SMOX for light-activated gas sensing applications.

## Methods

Flame spray pyrolysis was used for the thermophoretic deposition of the sensors. For preparation of the liquid feed, the appropriate amounts of precursors were dissolved in xylene, followed by homogeneous mixing in the ultrasonic bath for 10 min. During flame synthesis, premixed gas combination CH$_{4}$ + O$_{2}$ with 1.5 and 3.2 l/min, respectively, was combusted to obtain a pilot flame. Dispersion oxygen of 5 l/min was used for the fine dispersion of the feed liquid in the high-temperature flame for complete combustion. The nozzle tip was adjusted to 1.5 bar at these flow rates to realize fine particle production. Table 1 summarizes the deposition conditions and precursor chemicals used for synthesis of the sensors.

The sensors investigated in this work consist of an alumina substrate with interdigitated platinum electrodes. The back side of the substrates is equipped with a resistive heating structure [28]. For preparation of the flame-made sensors, the sensor substrates were mounted in a temperature-controlled sample holder above the FSP nozzle to maintain the substrate temperature at approximately 120 $^{\circ }$C during thermophoretic particle deposition. After deposition via FSP, the sensors were annealed in a tube furnace (Heraeus) at 400, 500, and 400 $^{\circ }$C, for 10 min each, followed by a cooldown until room temperature is reached [23, 29].

Sensor performance tests were carried out by mounting the such prepared sensors in a test chamber equipped with LEDs and electrical feed troughs. During all tests, the sensors were heated to 70 $^{\circ }$C. The gas atmosphere was provided by a fully automated gas mixing system with digital mass flow controllers and evaporators filled with deionized water. All gases and mixtures of pre-diluted analytes were provided by Westfalen AG. Resistances were recorded using a data acquisition unit (Agilent 34972A) with 100 MOhm precision resistors in parallel to the gas sensor. LEDs are operated at a constant forward current of 20 mA (Knick J152) [23]. The optical band gap was determined using diffuse reflectance UV–vis absorption spectroscopy using BaSO$_{4}$ as reference. The setup consists of a balanced halogen-deuterium lamp (Ocean Optics DH-2000-BAL), diffuse reflectance optics (Harrick Praying Mantis), and a UV–vis spectrometer (Ocean Optics Maya 2000 Pro). Reflectance spectra were transformed into Tauc plots for further evaluation.

## Results and discussion

### Material properties

The first step in the selection of materials for light-activated gas sensing is assessing their optical properties, most and foremost the optical band gap.

**Fig. 1:**
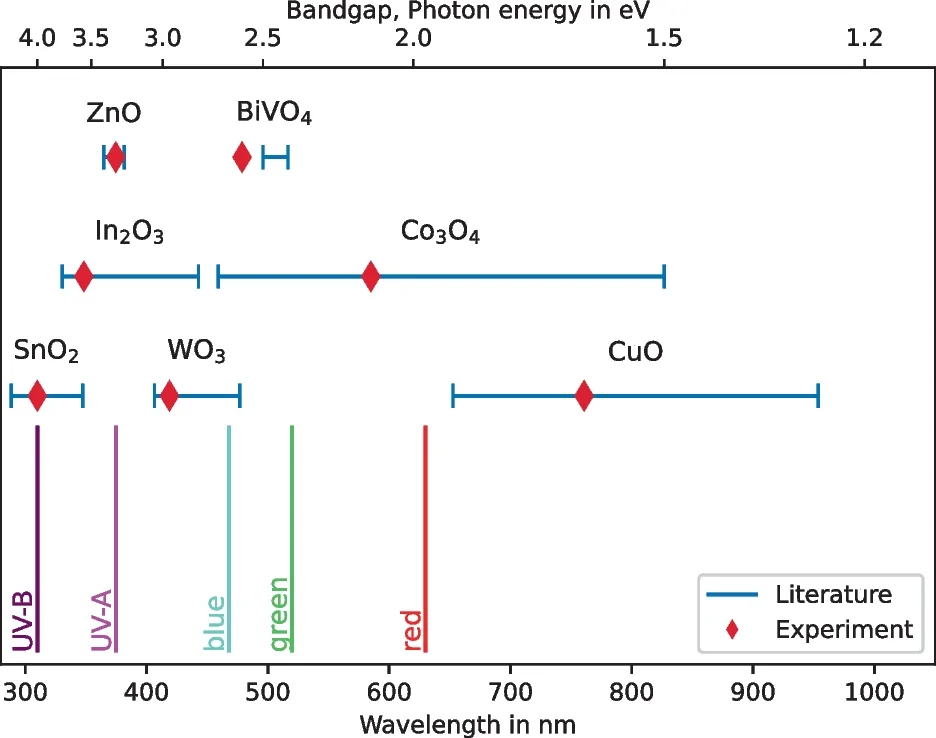
Experimental and reported values for the optical band gaps of semiconducting metal oxides as well as photon energies of the LEDs used in this study

Figure 1 illustrates the band gap energies of the SMOX as well as the photon energies of the LEDs used in this study. For the band gap, both reported and experimentally determined values are given [30–38]. The photon energies provided in the respective datasheets were validated with a spectrometer. For band to band excitation of the *p*-type semiconductors CuO, Co$_{3}$O$_{4}$, and BiVO$_{4}$, the photon energy of the red or green LEDs is sufficient whereas for the n-type materials, at least blue light (WO$_{3}$), UV-A (ZnO, In$_{2}$O$_{3}$), or even UV-B (SnO$_{2}$) is required. In case of Co$_{3}$O$_{4}$, multiple band gaps are reported [35].

**Fig. 2:**
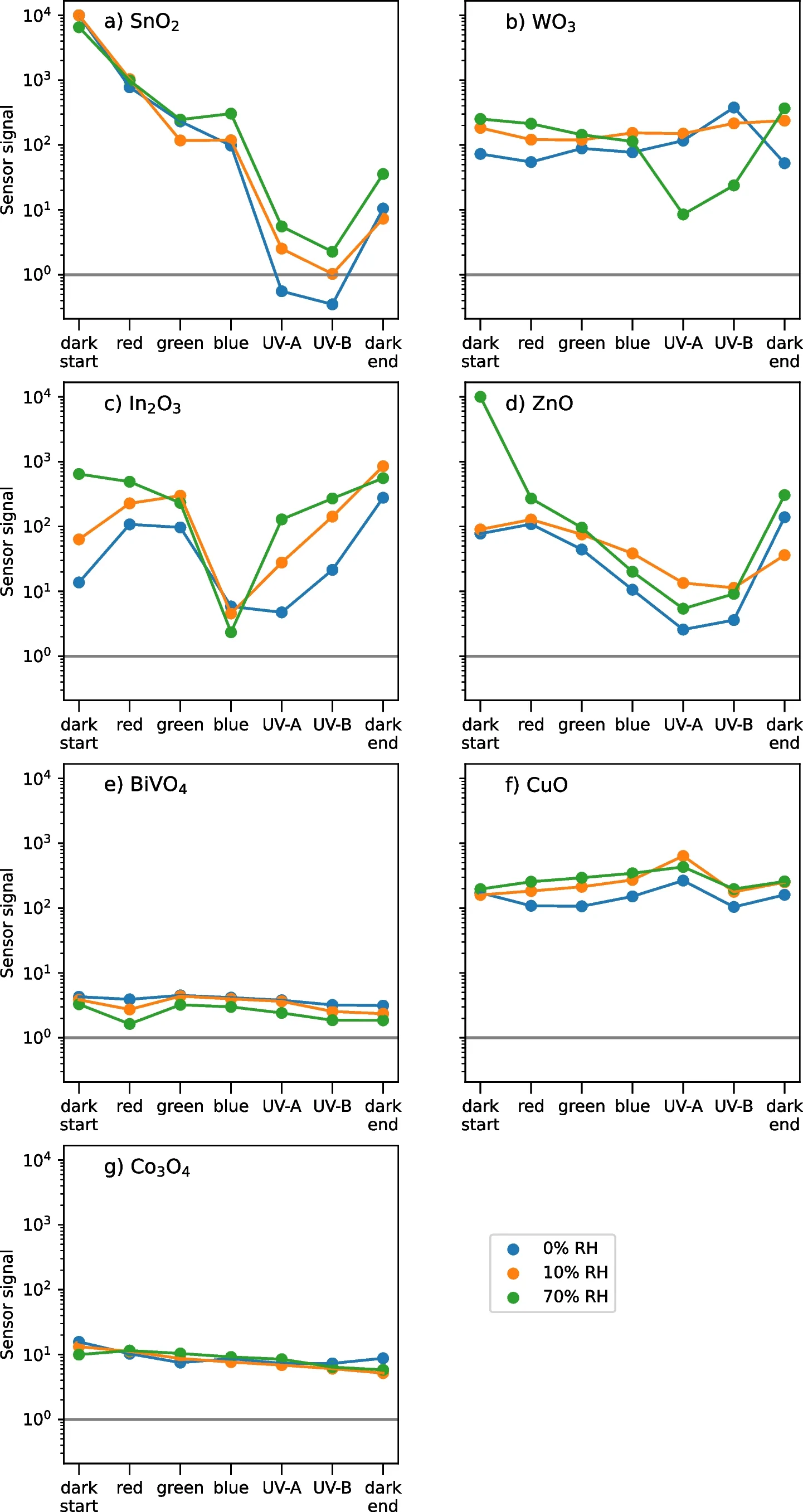
Sensor signals ($R_{\text {NO2}}/R_{\text {air}}$) of various materials at 70 $^{\circ }$C for the exposure to 0.5 ppm NO$_{2}$, obtained in different illumination conditions and background humidities

**Fig. 3:**
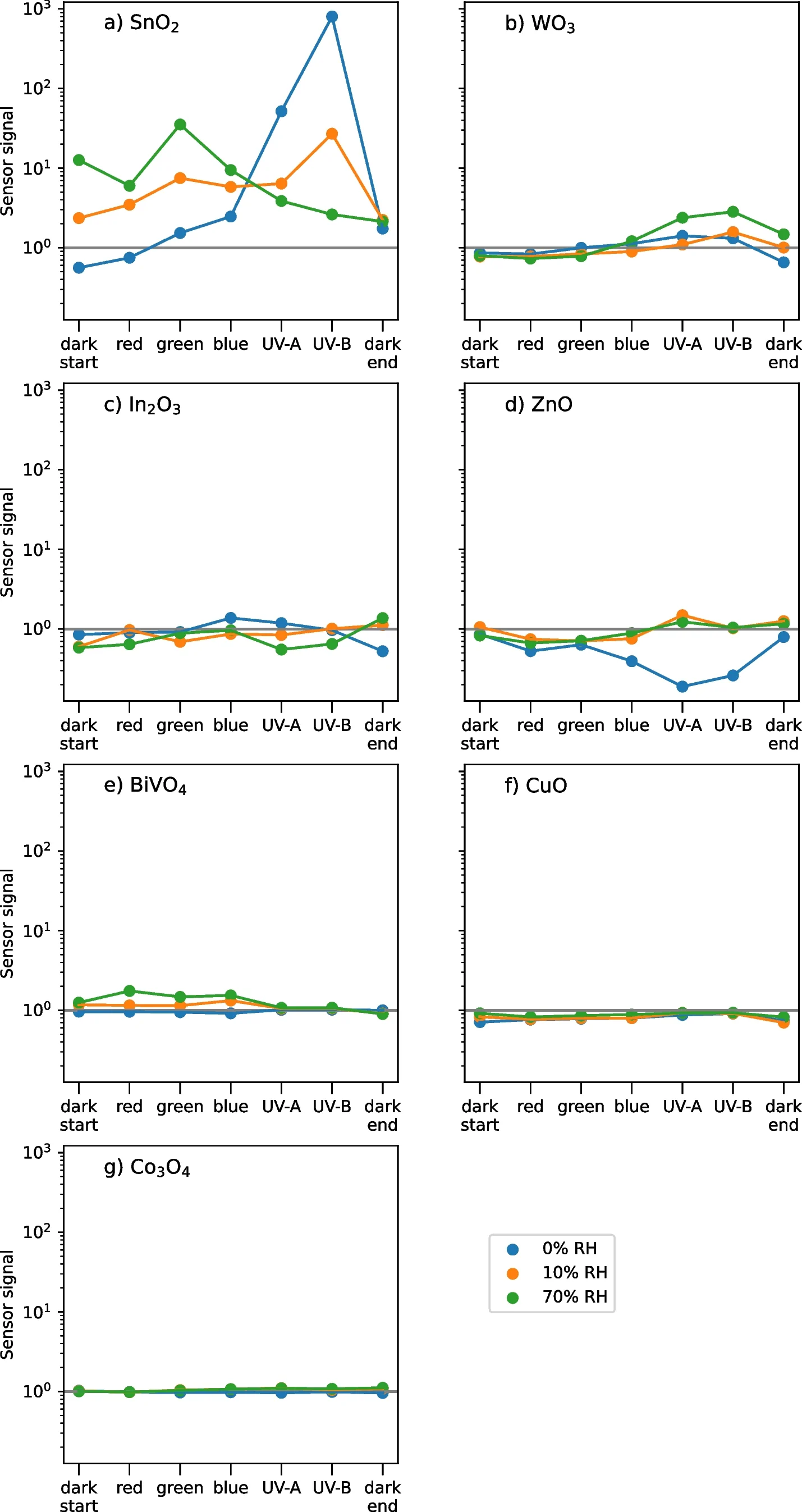
Sensor signals ($R_{\text {air}}/R_{\text {CO}}$) of various materials at 70 $^{\circ }$C for the exposure to 100 ppm CO, obtained in different illumination conditions and background humidities

**Fig. 4:**
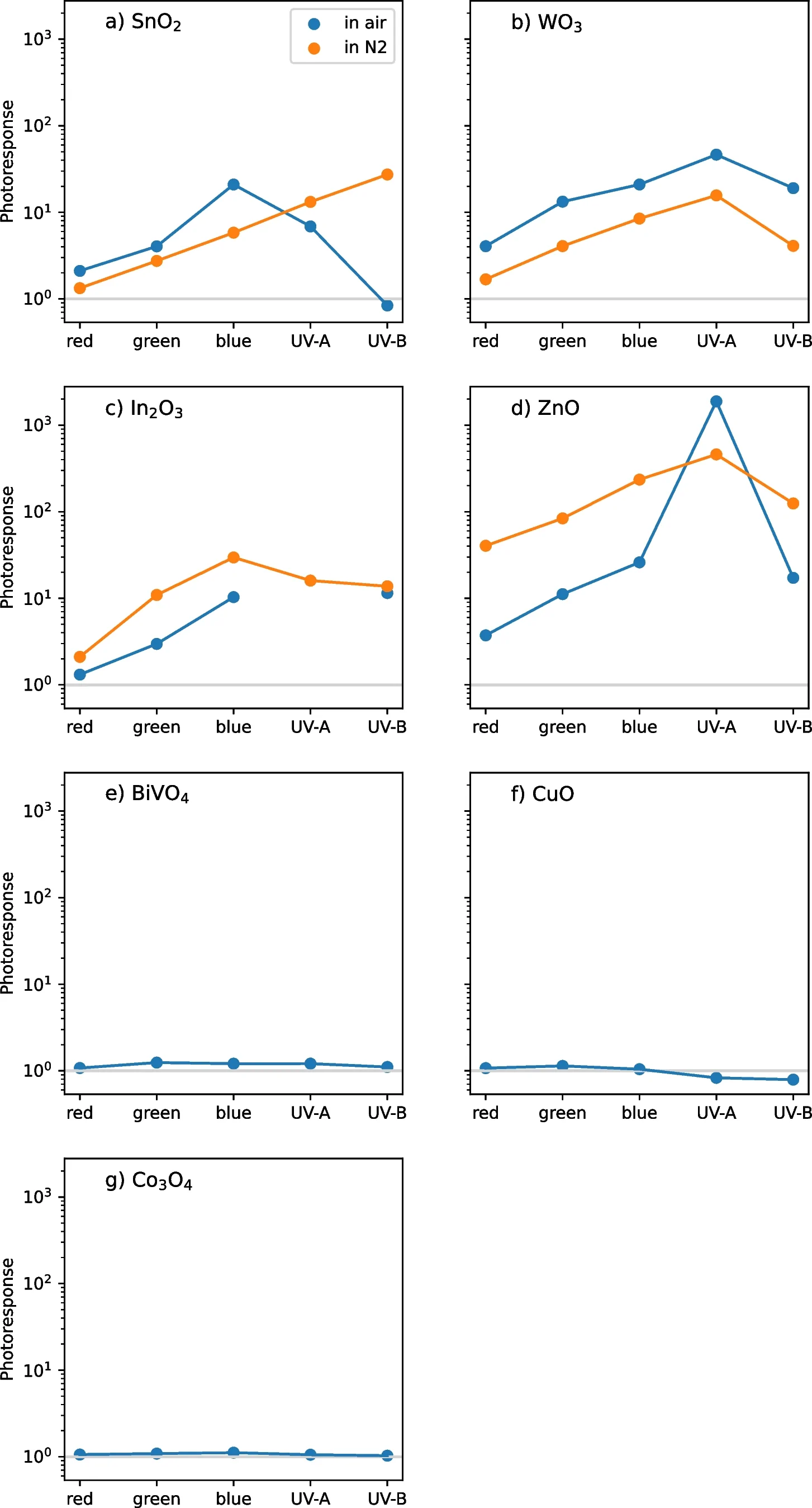
Sensor signals ($R_{\text {dark}}/R_{\text {light}}$) of various materials at 70 $^{\circ }$C for the exposure to light in dry air (blue) and dry nitrogen (orange)

### Gas sensing performance

The detection of NO$_{2}$ requires only little energy and is possible already at room temperature for certain materials. In this study, the SMOX sensors are operated at a slightly higher temperature of 70 $^{\circ }$C. With all materials presented in Fig. 2, NO$_{2}$ is detected in the absence of light, in many cases with even higher signals than under illumination.

**Fig. 5:**
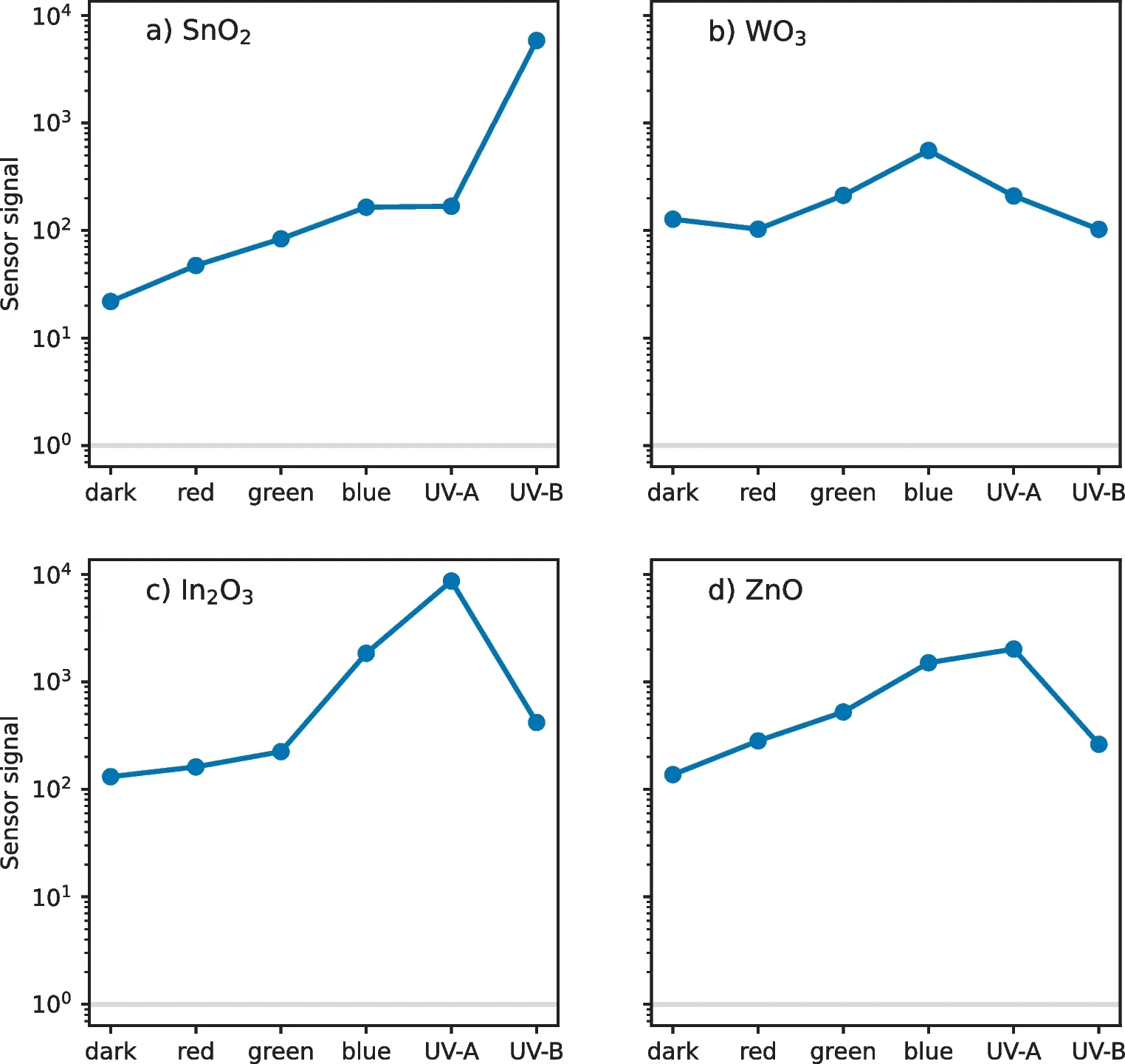
Sensor signals ($R_{\text {air}}/R_{\text {N2}}$) of various materials at 70 $^{\circ }$C for the transition from pure N$_{2}$ to synthetic air (79.5% N$_{2}$, 20.5% O$_{2}$)

**Fig. 6:**
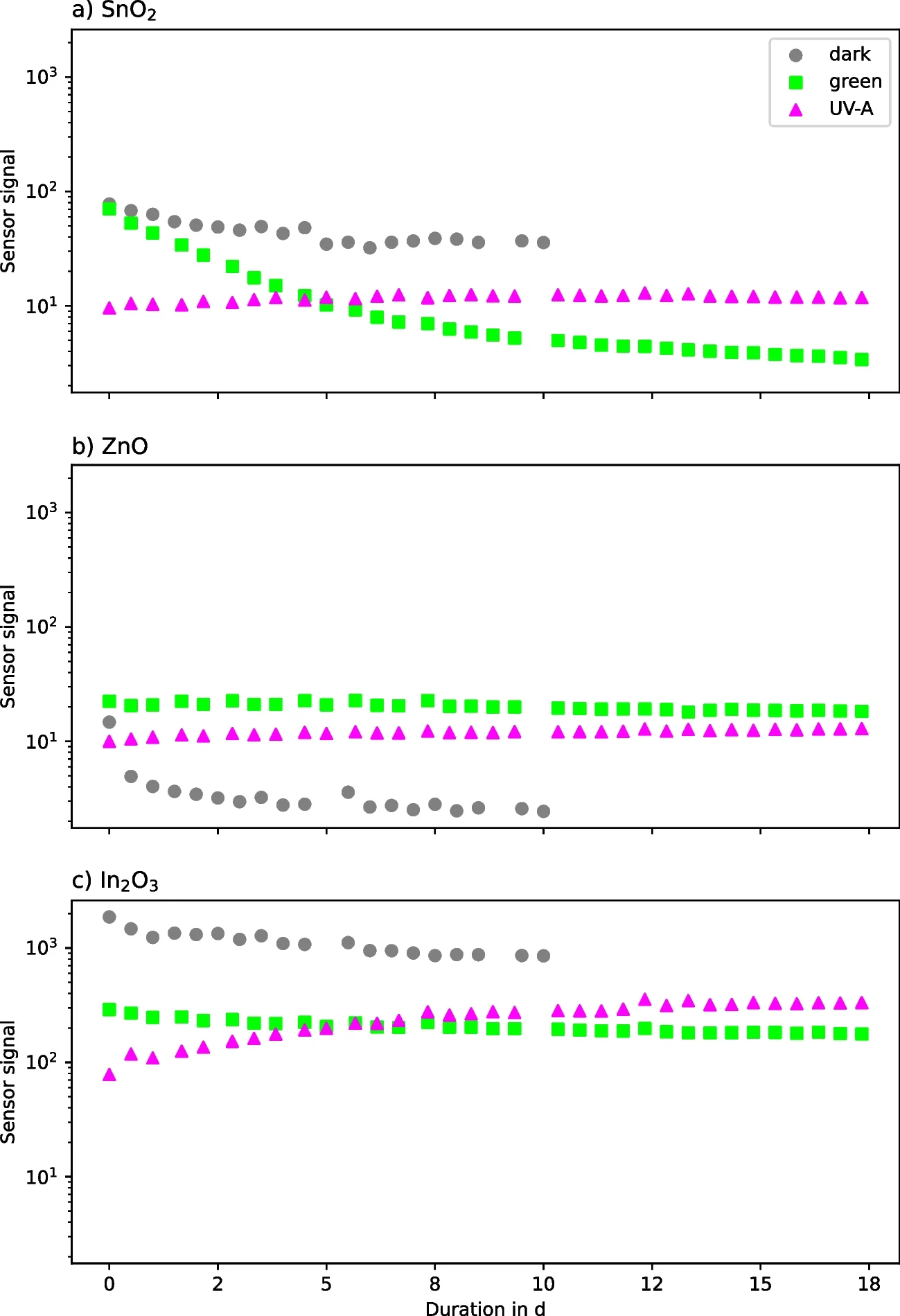
Sensor signals for the repeated exposure of SnO$_{2}$, ZnO, and In$_{2}$O$_{3}$ to 0.5 ppm NO$_{2}$ in a background of 30% RH

The highest dependency on wavelength is observed for SnO$_{2}$: In the visible range (red, green, blue), remarkably high sensor signals are observed, and NO$_{2}$ clearly has an oxidizing effect on SnO$_{2}$, characterized by the increase in resistance. The situation is rather different when UV light is introduced: The signals are lower, and the exposure to NO$_{2}$ results in a decrease in resistance in 70% RH. After these tests, another experiment was performed in the absence of light with the same sensors. Even though a reducing effect of NO$_{2}$ was observed again, the signals were not as high as initially. This is a clear indication that the exposure to the analytes under UV light is not fully reversible. In contrast to SnO$_{2}$, the response of WO$_{3}$ to NO$_{2}$ is independent not only from the wavelength, but also from the presence or absence of light. The sensor signals remain constant throughout all conditions, except for some lower signals under UV-light exposure. The signals obtained on In$_{2}$O$_{3}$ again show good response in darkness or under illumination with red or green and, depending on the humidity, slightly lower signals under UV-light illumination. Interestingly, under blue light illumination, the sensor signals are much lower. With ZnO, the sensor signal decreases gradually as photon energy increases. Of all materials, BiVO$_{4}$ shows the lowest sensor signals, which are neither increased nor diminished by illumination. CuO shows high sensor signals, whereas the sensor signals of Co$_{3}$O$_{4}$ are almost as low as for BiVO$_{4}$.

Carbon monoxide was chosen as a representative for reducing gases. One reason is its rather simple molecular structure and therefore lower prospect of possible chemical reactions, and another one lies in its ability to react with surface oxygen to form CO$_{2}$ without prior dissociation of chemical bonds. Nevertheless, the detection of CO is not possible at all with CuO or Co$_{3}$O$_{4}$ at 70 $^{\circ }$C, regardless of illumination (see Fig. 3). Also for BiVO$_{4}$ and In$_{2}$O$_{3}$, low sensor signals under illumination at only certain wavelengths can be observed. WO$_{3}$ and ZnO respond only at high photon energies. Interestingly, for WO$_{3}$, the resistance decreases, which is expected for a reducing analyte interacting with an *n*-type semiconductor, while for ZnO, the resistance increases. SnO$_{2}$ is the only material among the tested ones where high responses to CO are observed, even in the absence of light. Depending on the background humidity, the signal decreases (70% RH) or increases over multiple decades (0% RH) with photon energy.

In summary, these results show that the gas sensing properties under illumination are vastly different for each SMOX. For none of them, a substantially different behavior could be found when the photon energy of the illumination exceeds the band gap. Also, humidity will affect the sensor response qualitatively and quantitatively.

Concerning their sensor performance with respect to different analytes, for the detection of CO, the only responsive material among the tested ones is SnO$_{2}$. For NO$_{2}$, it also provides the highest sensor response under visible light illumination or in the absence of light. However, if the application is asking for independence from the wavelength, CuO is better suited due to its high signals and its tolerance for changes in humidity.

### Interaction with light

The interaction of a material with light itself is quantified in two independent experiments, presented in Fig. 4: For one, the LED is turned on while the sensor is operated in a background of dry air (20.5% O$_{2}$), while for the other, the sensor is kept in dry nitrogen. The signal *S* (or photoresponse) is calculated from the resistances in the respective conditions as $S=R_{\text {dark}}/R_{\text {light}}$. For all materials, the signals for red, green, and blue light increase as the photon energy of the light increases. Higher photon energies allow the excitation of charge carriers from additional states, which further increases the concentration of free charge carriers. At higher photon energies, the materials behave differently: For SnO$_{2}$, the photoresponse decreases while for ZnO and WO$_{3}$, there is a maximum at UV-A. This might be due to the interplay of photon energy and irradiance. Because the latter decreases going from blue to UV-A and UV-B, less free charge carriers are created in SnO$_{2}$. For ZnO and WO$_{3}$, on the other hand, UV-A matches or exceeds the band gap energy, and while fewer photons are provided, they are able to activate band-to-band excitation in these materials. The behavior of In$_{2}$O$_{3}$ under UV-A irradiation is special as the resistance first decreases, but then increases over time, ending up higher than the baseline in darkness. For the low band gap materials, almost no photoresponse is observed. This explains in part why the response of these materials to NO$_{2}$ is independent from the illumination.

Besides the immediate effect of creating additional charge carriers, the photodesorption of oxygen must also be considered. To separate these effects, the same experiment is repeated in nitrogen instead of air. Here, the desorption of oxygen is irreversible, as only traces of O$_{2}$ are available. Therefore, one would expect higher photoresponse in N$_{2}$ compared to air, even though there is already less oxygen at the surface in dark conditions. For ZnO, In$_{2}$O$_{3}$, and WO$_{3}$, the same trends as in air are observed: increased photoresponse due to increased photon energy in the visible range, until the effect of the UV-B LED’s low irradiance prevails.

In a complementary series of experiments, presented in Fig. 5, the effect of oxygen was investigated. Except for the experiment in darkness, the sensor was kept in dry nitrogen under illumination until the resistance was stable before 20.5% oxygen was introduced, corresponding to dry air. For SnO$_{2}$, the largest increase in resistance was observed with UV-B illumination; also, the other materials showed the highest sensor signals at photon energies just above their band gap (blue for WO$_{3}$ and UV-A for ZnO and In$_{2}$O$_{3}$). For UV-B, the signals decrease. This is remarkable, as this is the only experiment in this study where the effect of the band gap became apparent.

Both O$_{2}$ and NO$_{2}$ are oxidizing gases, yet, because different chemical reactions are involved in their reception, the dependency of their response on wavelength is highly different: On SnO$_{2}$, signals are increasing with photon energy for O$_{2}$, but decreasing for NO$_{2}$. This is a clear indication of how illumination can be used to modify a material’s selectivity.

When the photon energy exceeds the band gap, the charge carrier density in the conduction band is increased via band-to-band excitation. The chemisorption of oxygen at the surface is probably the most fundamental reaction for gas sensing with SMOX [8, 39]:

1 $$\begin{aligned} 1/2 \text {O}_{2} +2~\text{ e}^{-} <=> \text {O}^{2-} \end{aligned}$$

This reaction requires two electrons. If more electrons are available, the equilibrium is shifted towards the products, which explains the higher sensor signals above the band gap energy. Below, electrons are only available thermally or excited from band gap states by visible light and therefore lower in concentration. For UV-B, the low photon flux is again the restrictive quantity.

At first glance, these findings are contradictory to the photoresponses presented in 4. For example, ZnO under UV-A illumination shows the highest sensor signal for oxygen (i.e., increase in resistance), but also the highest photoresponse in air (decrease in resistance). Therefore, we conclude that illumination affects the adsorption and desorption of oxygen independently and therefore also the state of the surface is dependent on the sequence of the previous stimuli such as illumination or gas exposure. Also, the response to oxygen is significantly higher than the photoresponse.

### Long-term stability

Long-term stability is a key factor for the operation of sensors. In order to demonstrate the feasibility of these sensors for application, an experiment with repeated exposure to 0.5 ppm NO$_{2}$ in a background of 30% RH has been performed. The illumination conditions chosen were dark, green light, whose photon energy is below the band gap of all materials, and UV-A, where qualitatively different sensor performance was observed. The other materials were omitted from these tests since their dependency on wavelength is less pronounced. Figure 6 shows the development of the sensor response over the course of two weeks. Even though good sensor response is observed for dark and green illumination conditions, its performance is drastically decreased over time. In contrast, the low signals obtained under UV-A illumination are stable. ZnO shows rather low, but stable signals for both LEDs, but lacks stability in darkness. In$_{2}$O$_{3}$ provides rather stable signals in all illumination conditions, with a slight increase for UV-A. The long-term investigation clearly shows that stable operation is highly dependent on the individual combination of SMOX and illumination. Therefore, no clear rules for the selection of materials can be devised without prior investigations and tests related to the individual application.

## Conclusion

The gas sensing performance of seven different SMOX was compared under illumination.

In general, the interaction of the surface with oxygen is highly affected by illumination, and both adsorption and desorption reactions are dependent on the wavelength. The results also imply that band-to-band excitation of electrons affects the chemisorption of oxygen, but its impact on the gas sensing performance itself is often negligible.

Yet, for each material, specific observations were made: SnO$_{2}$ is most affected by illumination, even to the extent of reversing the direction of the sensor response. Also, it is the only material which is able to detect CO. This makes it the most promising material for light-activated gas sensors, but the underlying mechanisms need to be investigated further. The signals of ZnO show correlation with wavelength, and therefore, ZnO has high potential for tuning the selectivity. Also, for In$_{2}$O$_{3}$, a high dependency on the wavelength was observed; however, the signals are not in line with photon energy. The impact of illumination is least pronounced for WO$_{3}$.

On the one hand, the dependency on illumination is an opportunity to tune the sensor’s properties such as selectivity, sensitivity, and baseline resistance and therefore allows the extraction of additional information from the same sensing layer when different illumination conditions are applied. On the other hand, the mechanisms responsible for the dependency on wavelength need to be understood well to ensure reliable operation of the sensors outside laboratory conditions.

While low band gap semiconductors seem to offer easy activation with visible light, they were barely affected by illumination at all. Therefore, established wide band gap SMOX like SnO$_{2}$ or ZnO are preferred materials also under illumination. However, their stability needs to be assessed in long-term studies, closely related to the intended application. Before and after experiments under illumination, the sensors’ performance was assessed in dark conditions. The deviation between these experiments, especially for SnO$_{2}$, also highlights the need for these kinds of tests.

Some of the most interesting aspects in this exploratory study such as the behavior of In$_{2}$O$_{3}$ under blue light illumination or the oxidizing effect of CO on ZnO require further insights and will be the objectives of follow-up studies using operando spectroscopic methods [40]. Also, for assessing the selectivity of light-activated gas sensors, more relevant oxidizing and reducing analytes, e.g., ozone and hydrogen, will be employed [4, 9, 41].

## Data Availability

Data are available from the corresponding author upon reasonable request.
